# Endovascular Repair of Ruptured Abdominal Aortic Aneurysm in COVID 19 Pandemic

**DOI:** 10.1007/s44229-022-00003-0

**Published:** 2022-06-01

**Authors:** Lubna Alrowaili, Alvaro Balcazar, Owayed Al Shammeri

**Affiliations:** Alrayyan Hospital, Habib Medical Group, Riyadh, Saudi Arabia

**Keywords:** Abdominal aortic aneurysm, Ruptured abdominal aortic aneurysm, EVAR

## Abstract

Abdominal aortic aneurysms are pathological dilatations of the abdominal aorta that occur because of unknown causes and are associated with cardiovascular risk factors excluding diabetes. Ruptured abdominal aortic aneurysms are catastrophic events with high mortality and morbidity. This case report describes successful management of a ruptured abdominal aortic aneurysm treated with endovascular aortic repair through the bell-button technique, with a short hospital course.

## Introduction

Abdominal aortic aneurysm (AAA) is a pathological dilatation of the abdominal aorta that is associated with adverse cardiovascular events and may rupture. Unfortunately, most patients with AAA are asymptomatic, and present for treatment after ruptures have caused massive internal bleeding—a condition with a high mortality rate of 80% [1]. Although the cause of AAA is unknown, the risk factors include cardiovascular factors but not diabetes. Advanced age and smoking are predominant risk factors. Men are three times more likely to be affected than women, and genetic factors are also important [2, 3]. The prevalence of AAA is 1–2% [4] worldwide, but a paucity of data exists in Saudi Arabia. A single screening study performed in the Asir region in a single center has indicated an AAA prevalence of 2.9% among 701 patients older than 60 years of age (AAA ≥ 3 cm, by ultrasound) [5]. The prevalence in men older than 65 years of age can reach 8% [6].

The mortality rate from ruptured AAA is extremely high, at 80% [1]. The relative benefits and advantages of endovascular repair (EVAR), compared with open repair, in the treatment of ruptured AAA has been well established in multiple clinical trials. Here, we discuss decision-making during the COVID-19 pandemic in a case with unsuitable anatomy, and describe how we overcame the challenges successfully.

## Case presentation

A 71-year-old man presented to the emergency department with a 3-days history of abdominal pain, which was dull and aching, and had worsened on the day of presentation, scoring 7 out of 10 using universal pain assessment tool where zero is no pain and 10 is the worst possible pain. The pain radiated to the right iliac region and was not relieved by factors such as specific positioning. The patient denied any chest pain, syncope, or recent trauma. The patient’s past medical history included coronary artery disease after a coronary artery bypass graft in 2009. He had a cardiac catheter placed in 2016, with a patent left internal mammary artery graft to the left anterior descending coronary artery, and a saphenous vein graft to the right coronary artery. He had known end-stage renal disease and received regular hemodialysis via a right internal jugular vein permcath. He had chronic obstructive pulmonary disease (COPD). Despite having been an ex-smoker for more than 1 year, he had a 100 pack-year history of smoking. He also had severe hearing loss that limited direct communication.

**Fig. 1:**
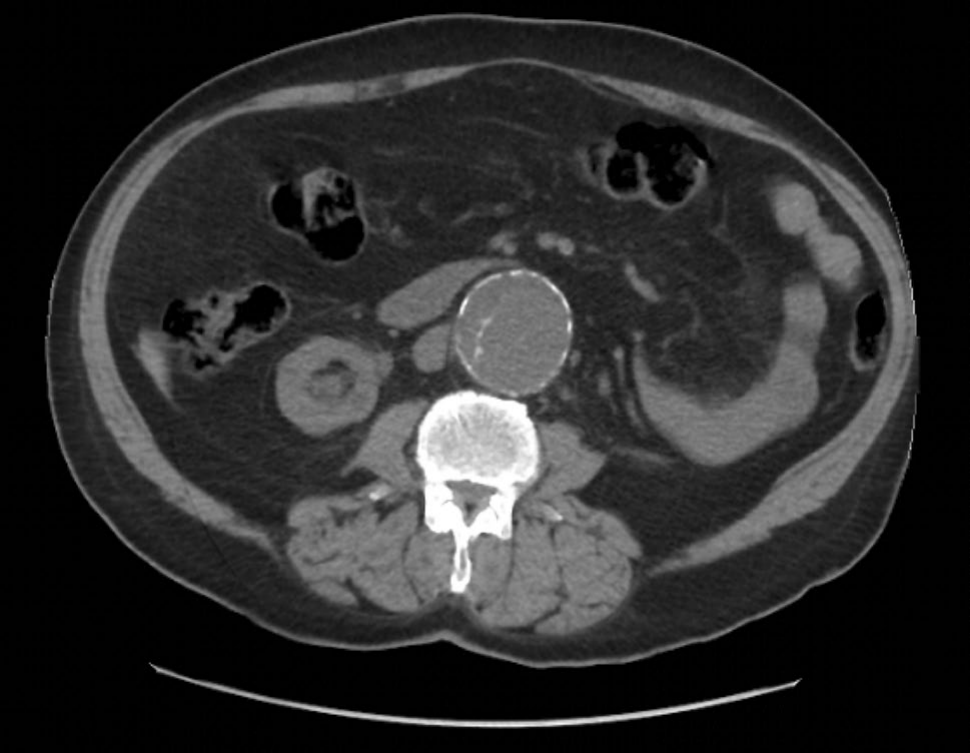
CT scan from 2017 with contrast illustrated infrarenal abdominal aortic aneurysm with maximum diameter of 5 cm

He had a previously known case of AAA, which was discovered incidentally in October 2017 during abdominal imaging. The findings at that time, through computed tomography angiography (CTA) of Aorta are, extensive atherosclerotic disease with infrarenal AAA diameter of 5 cm (Fig. 1). There is loss of follow up and communication barrier increasing with time due to hearing loss. Though, he had had a follow up CTA of aorta in May 2018 showed no significant interval change as compared to the previous CTA October 2017.

Examination revealed a blood pressure of 124/56 mm Hg, heart rate of 90 beats per minute, temperature of 36.5 °C, and oxygen saturation of 98% on room air. The patient appeared pale and in mild distress. Abdominal examination revealed a slightly distended abdomen with mild generalized tenderness and no guarding.

**Fig. 2:**
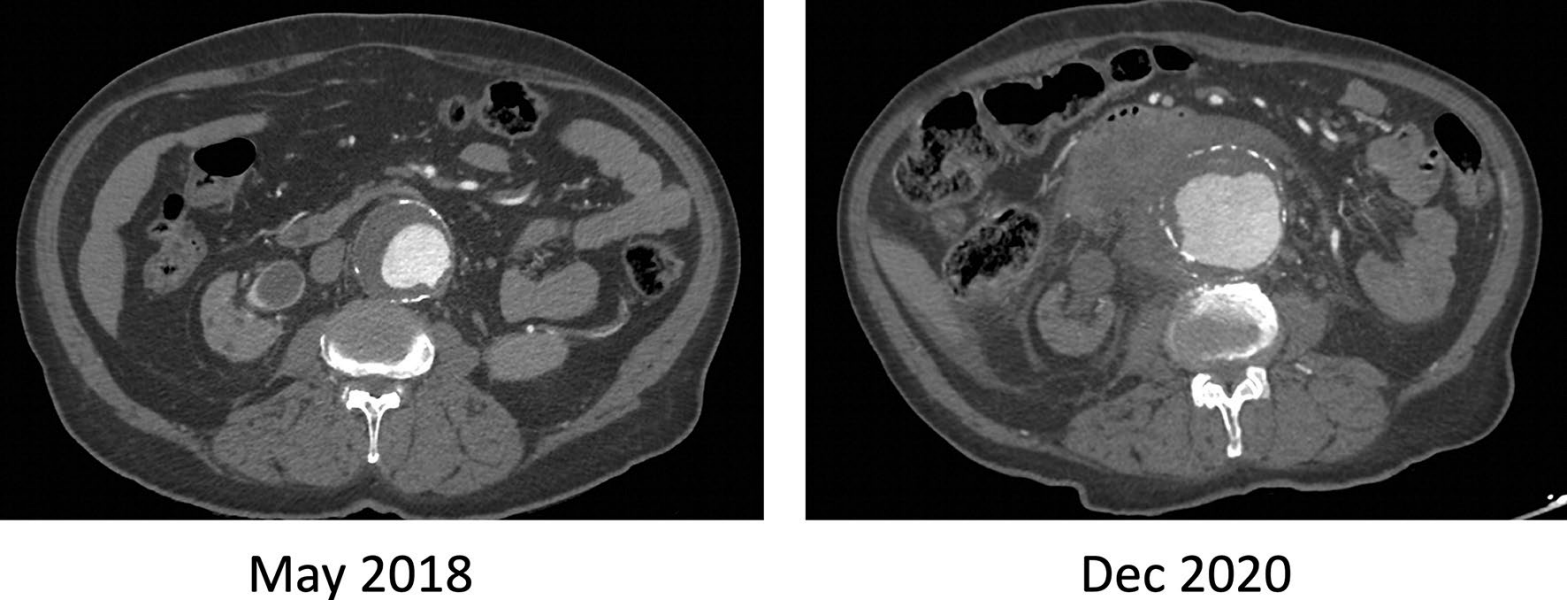
CT scan with contrast comparing 2017 and 2020 showed marked expansion of abdominal aortic aneurysm with evidence of retroperitoneal bleeding

**Fig. 3:**
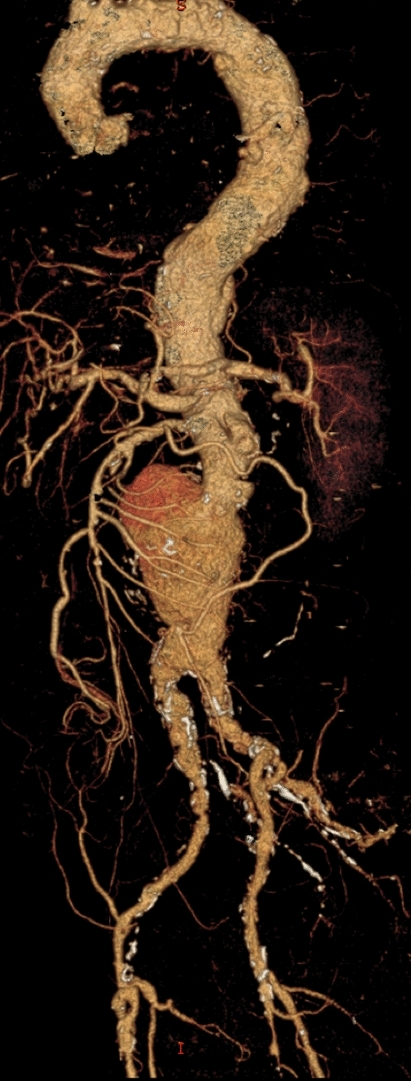
3-D CT aortogram reconstruction showed the lateral bulge of the abdominal aortic aneurysm and severely diseased and ectatic aorta

Complete blood count indicated profound anemia (hemoglobin 5.5 g/dl) and mild leukocytosis (white blood cell count 14). The platelet count was 191 × 10^3^/μl. An emergency CT scan with contrast showed a contained AAA rupture. The AAA diameter increased to 7.7 cm with a luminal bulge at the superior right lateral aspect, with adjacent moderate acute retroperitoneal hematoma displacing the third part of the duodenum (Figs. 2 and 3).

Access through two large pore IVs was established, and analgesic was administered. Four units of cross-matched packed red blood cells were transfused. An emergency multidisciplinary meeting among intensive care, anesthesia, vascular surgery, and interventional cardiology teams was held. Emergency intervention was determined as mandatory to save life. The benefits and drawbacks of open surgery versus EVAR were discussed. Given the patient’s comorbidities (heart failure, severe peripheral vascular disease, COPD, end-stage renal disease on hemodialysis, and advanced age), a final decision was made to manage the ruptured aneurysm with emergent EVAR. The challenges of EVAR, as addressed in the meeting, were as follows: (1) the largest abdominal stent graft system was 36 mm, and the smallest diameter away from the aortic hematoma was 40 mm; (2) the aortic wall was diffusely diseased, with severe atherosclerotic plaques, dissections, and ulcerations, and the largest focal ulceration was at the level of the superior mesenteric artery (SMA); (3) a left internal mammary graft was present with diseased aortic great vessels and a type three aortic arch, thus making the chimney procedure to the SMA and celiac axis very challenging and hazardous to the blood supply via the left internal mammary artery graft, because the SMA and celiac diameters exceeded 9 mm; (4) this was an emergency case with a high likelihood of mortality, thus requiring an immediate solution.

**Fig. 4:**
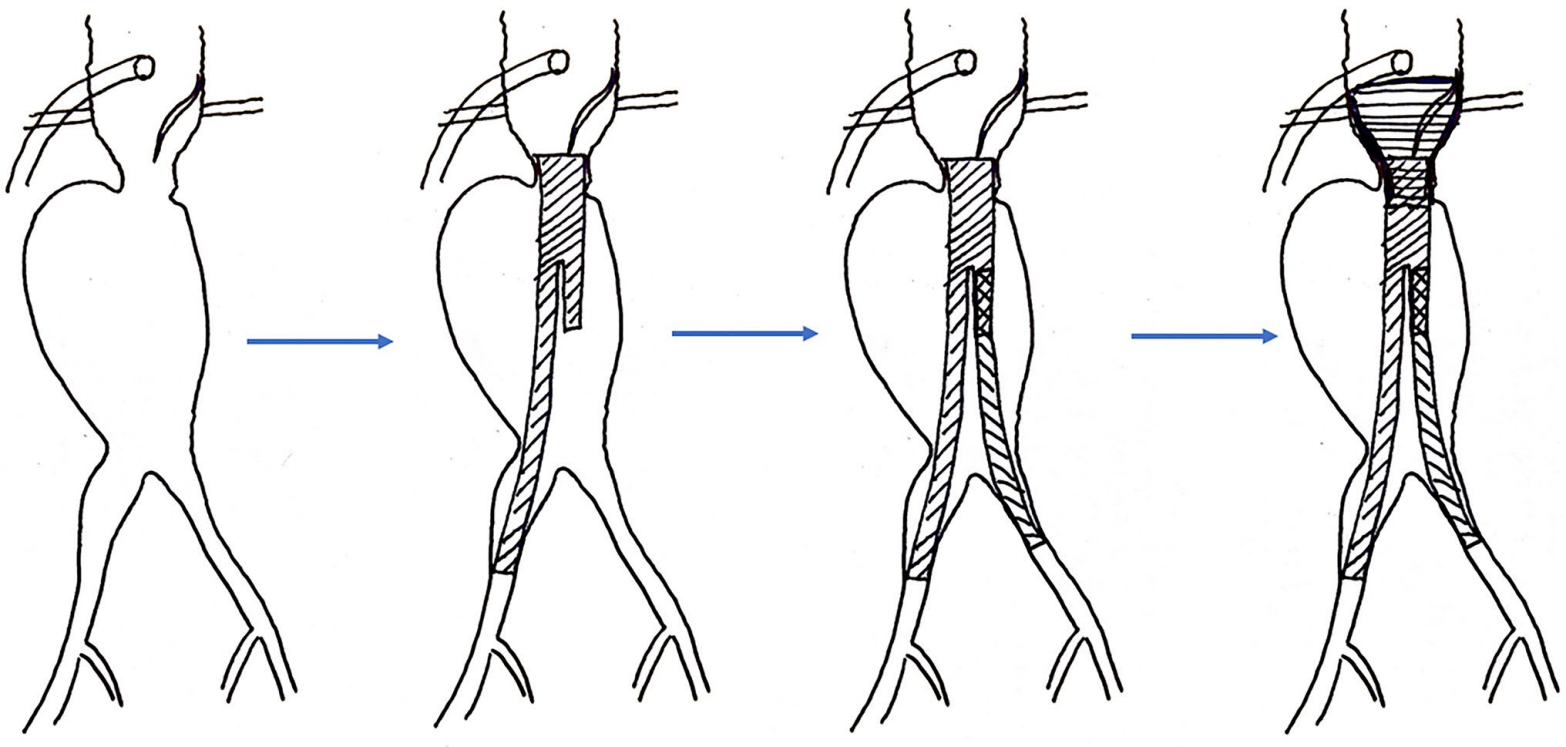
Schematic diagram of EVAR procedure steps

**Fig. 5:**
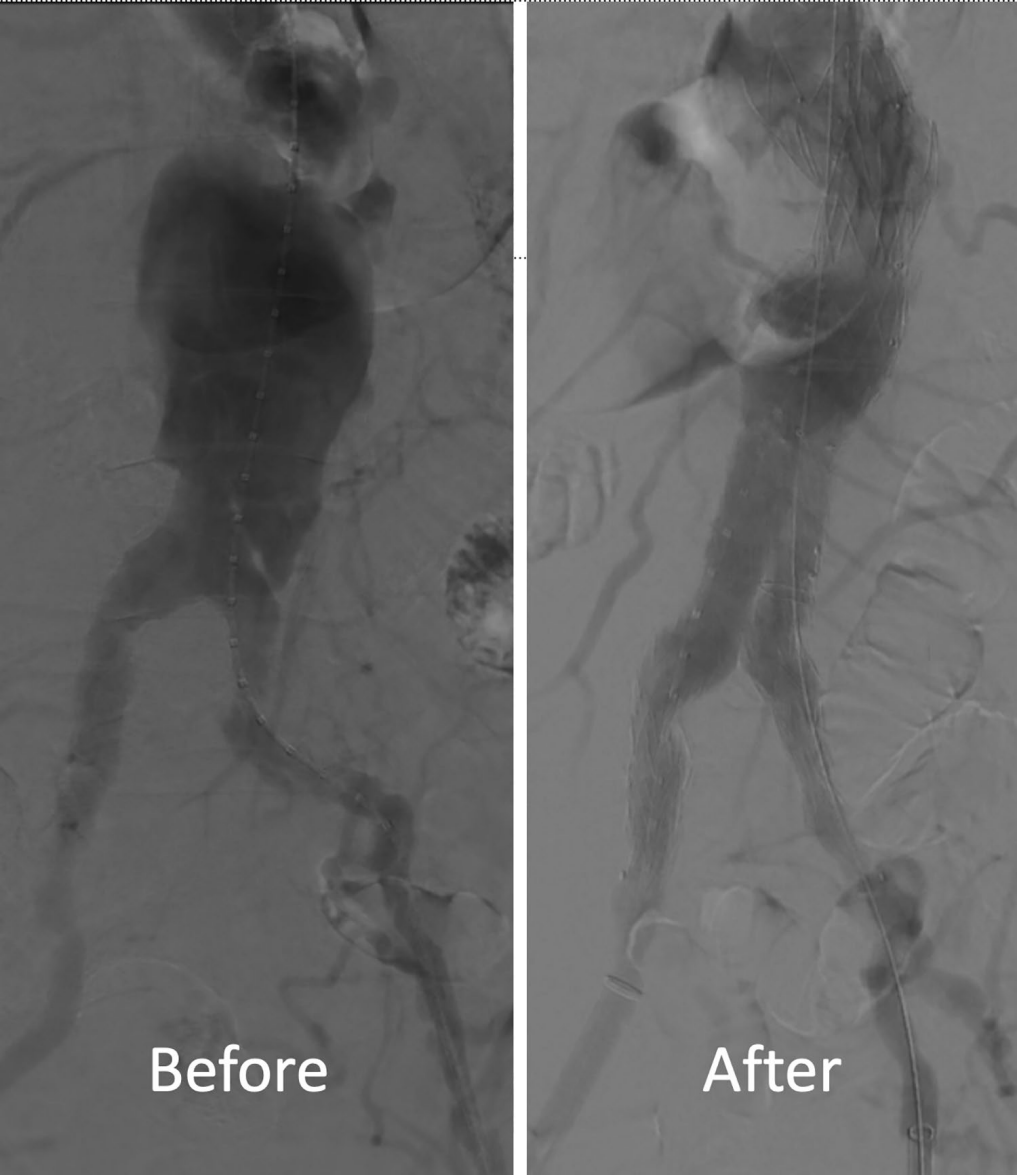
Pre- and post-EVAR angiogram with digital subtraction

The patient was taken to the catheterization laboratory on the same day and underwent successful emergency EVAR (strategy shown in the schematic in Fig. 4). We used an EVAR body graft with 36 mm proximal diameter, 20 mm distal diameter, and 166 mm length, and a contralateral graft with 16 mm proximal diameter, 13 mm distal diameter, and 126 mm length. We then used a short thoracic EVAR with 43 mm proximal and distal diameters, and 62 mm length, serving as a proximal cuff covering the renal arteries, covering the neck with aortic hematoma, and sealing the large aortic dissection in the juxtarenal region (before and after angiographic images in Fig. 5). The procedure was performed percutaneously, and Proglide closure devices were used in the groin on both sides successfully. The patient was extubated in the catheterization laboratory and then moved to the ICU in good condition. No further decrease in hemoglobin was observed, and the patient was ambulating the next morning. Three-day monitoring in the hospital was uneventful, and the patient was discharged home.

## Discussion

Ruptured AAA is associated with high morbidity and mortality, reaching 80%, but the feasibility of immediate intervention is challenged by little time available for accurate planning and sizing, and frequent nonoptimal endograft size [7]. This case illustrates how to “think outside the box” by using a thoracic EVAR prosthesis in the AAA and sealing renal arteries in a patient who already had nonfunctioning kidney organs. Another challenge was that this case required immediate intervention during the COVID-19 pandemic. Fortunately, rapid COVID-19 testing was available, and the results were obtained in less than 12 h. We used a bell-bottom technique to simplify the EVAR procedure, because the common iliac arteries are less than 20 mm in diameter. The bell-bottom technique is the most commonly performed procedure to preserve the hypogastric artery, but it requires no more than 20 mm common iliac artery aneurysm, given the elevated risk of type 1b endoleak (from 2.2 to 18%) [8]

In general, open surgery may be preferable in cases of ruptured AAA with complex anatomy. However, in light of this patient’s comorbidities (particularly COPD and ischemic heart disease), we performed emergency EVAR. In a recent meta-analysis, after risk adjustment, the 30-days mortality has not been found to differ between open surgical repair and EVAR in ruptured AAA [9]. In another meta-analysis including only randomized controlled trials with a low overall risk of bias, no difference has been found in short-term outcomes (30-days mortality) between emergency EVAR and emergency open repair [10]. In this meta-analysis, only three randomized controlled trials reported endoleak, and a total of 44 endoleak events occurred in 128 participants randomized to emergency EVAR treatment. In our patient, no endoleak occurred. The patient refused a postprocedure CT scan to rule out late type I endoleak, but he was clinically stable with regular follow up in the hemodialysis unit. Notably, the European Society for Vascular Surgery (ESVS) 2019 Clinical Practice Guidelines on the Management of Abdominal Aorto-iliac Artery Aneurysms [11] recommend 30% graft oversizing to prevent late endoleak in patients with hypovolemic shock, but in our case, the patient was not in shock.

The COVID-19 pandemic modified our treatment strategies across all specialties. With the aim of achieving the best possible medical management, emergency EVAR allowed us not only to treat this patient with complications but also to expedite the discharge, thus making an extra bed available for another patient needing intensive care. In terms of ethical considerations, the management of emergency cases still must consider safety, even in the COVID-19 pandemic. Although the anatomical challenges did not lead to adverse outcomes in this case, other cases could have different outcomes. This case report may serve as a reference for related clinical management. However, advanced validation must be conducted before future clinical application of this technique.

Maintaining follow-up despite the COVID-19 pandemic is crucial to decrease the rate of postoperative complications. The patient continually refused a controlled CT scan study, but ultrasound showed no endoleak. Clinically, the patient has remained asymptomatic and has no more pulsating mass in the abdomen.

## Conclusion

Although this is a single case report, it illustrates the value of emergency EVAR for ruptured AAA during the COVID-19 pandemic. The case had complex anatomy starting from severely diseased access, large diameter neck size and short neck length as well the bed crises management necessitating more Intensive care unit bed during COVID pandemic. A thoracic EVAR prosthesis was used, as no appropriate EVAR prosthesis for the neck was available in the market, to cover renal arteries in a patient with nonfunctioning kidneys to achieve an appropriate seal of a large aortic dissection in the juxtarenal region. The patient was ambulating 12 h after the procedure and was discharged home after 3 days of clinical monitoring.

## Data Availability

The data that support the findings of this study are available from the corresponding author (OA) upon reasonable request.
